# Supplementary material for: A TrkB and TrkC partial agonist restores deficits in synaptic function and promotes activity‐dependent synaptic and microglial transcriptomic changes in a late‐stage Alzheimer's mouse model
Source: Alzheimers Dement. 2024 May 23;20(7):4434–60. doi: 10.1002/alz.13857 (PMC11247716; doi:10.1002/alz.13857)
Supplement: Supplementary file 1 — Supporting Information [file ALZ-20-4434-s004.pdf]

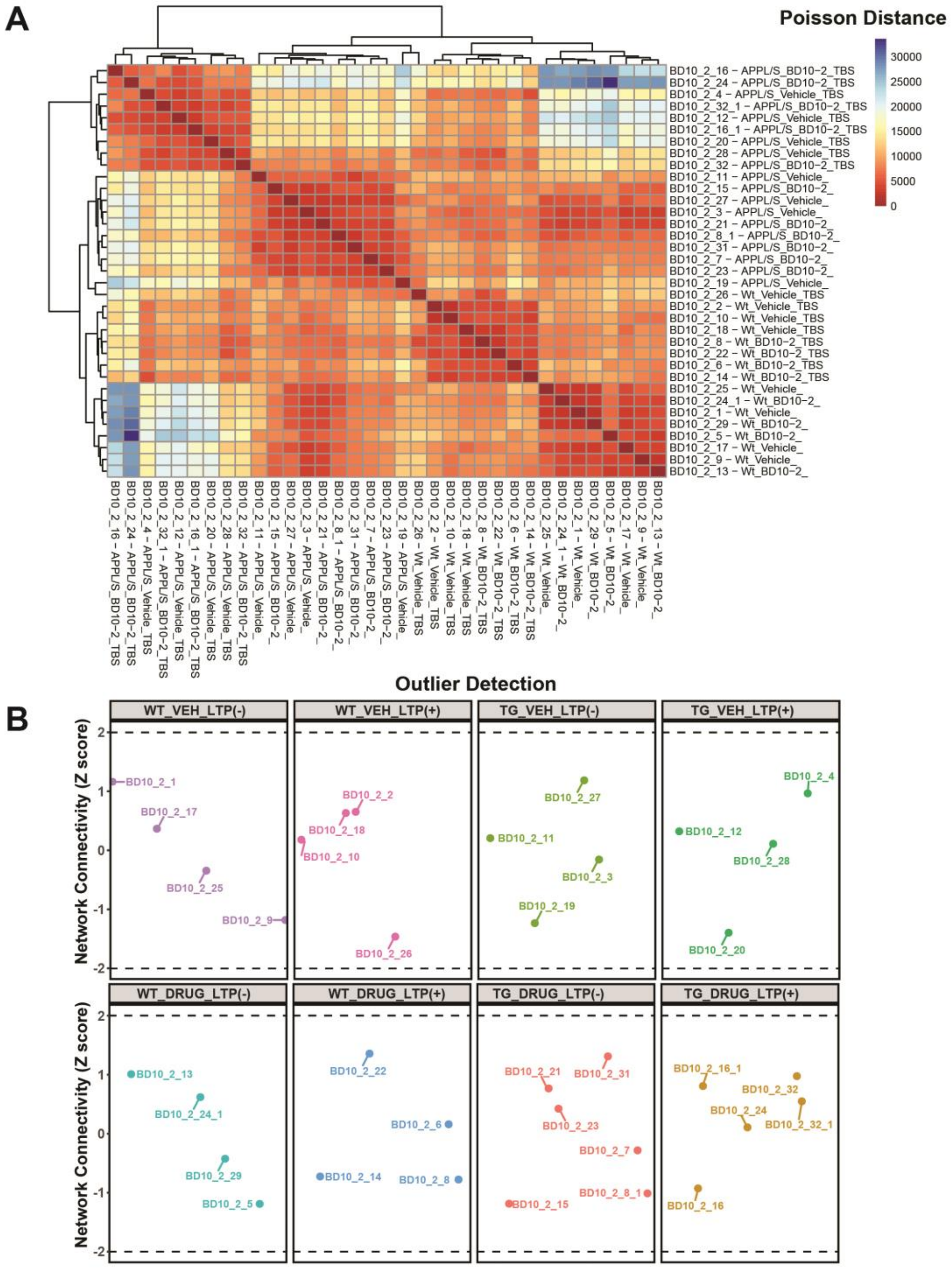

Supplementary Figure 1

Supplementary Fig. 1**. Effect of BD10-2 on proteins downstream of TrkB signaling in WT and APP$^{L/S}$ mice.** BD10-2 or vehicle was administered by oral gavage to 13-month-old APP$^{L/S}$ mice for three months, followed by collection of hippocampal slices. Western blots of hippocampal slice extracts were quantitated by determining the ratios of phospho (p) protein over total protein or total protein over actin, and then normalized to respective WT-Veh groups. Statistical significance was determined using either one way ANOVA with Sidak post hoc test or Kruskal-Wallis test with post-hoc Dunn's multiple comparisons test (mean ± SEM, Sample size = 6-10 hippocampal slices, 3-5 mice per group, with two independent western blots averaged per slice). **A-E** Quantification of TBS- hippocampal slice blots shown in **Fig. 4A**. **F.** Representative western blots for each TBS+ condition, except for GluA1 which is shown in **Fig. 4I**. **G-Q.** Quantification of TBS+ hippocampal slices shown in **Fig S1F** and **Fig 4I**.
