# Supplementary material for: A TrkB and TrkC partial agonist restores deficits in synaptic function and promotes activity‐dependent synaptic and microglial transcriptomic changes in a late‐stage Alzheimer's mouse model
Source: Alzheimers Dement. 2024 May 23;20(7):4434–60. doi: 10.1002/alz.13857 (PMC11247716; doi:10.1002/alz.13857)
Supplement: Supplementary file 2 — Supporting Information [file ALZ-20-4434-s007.pdf]

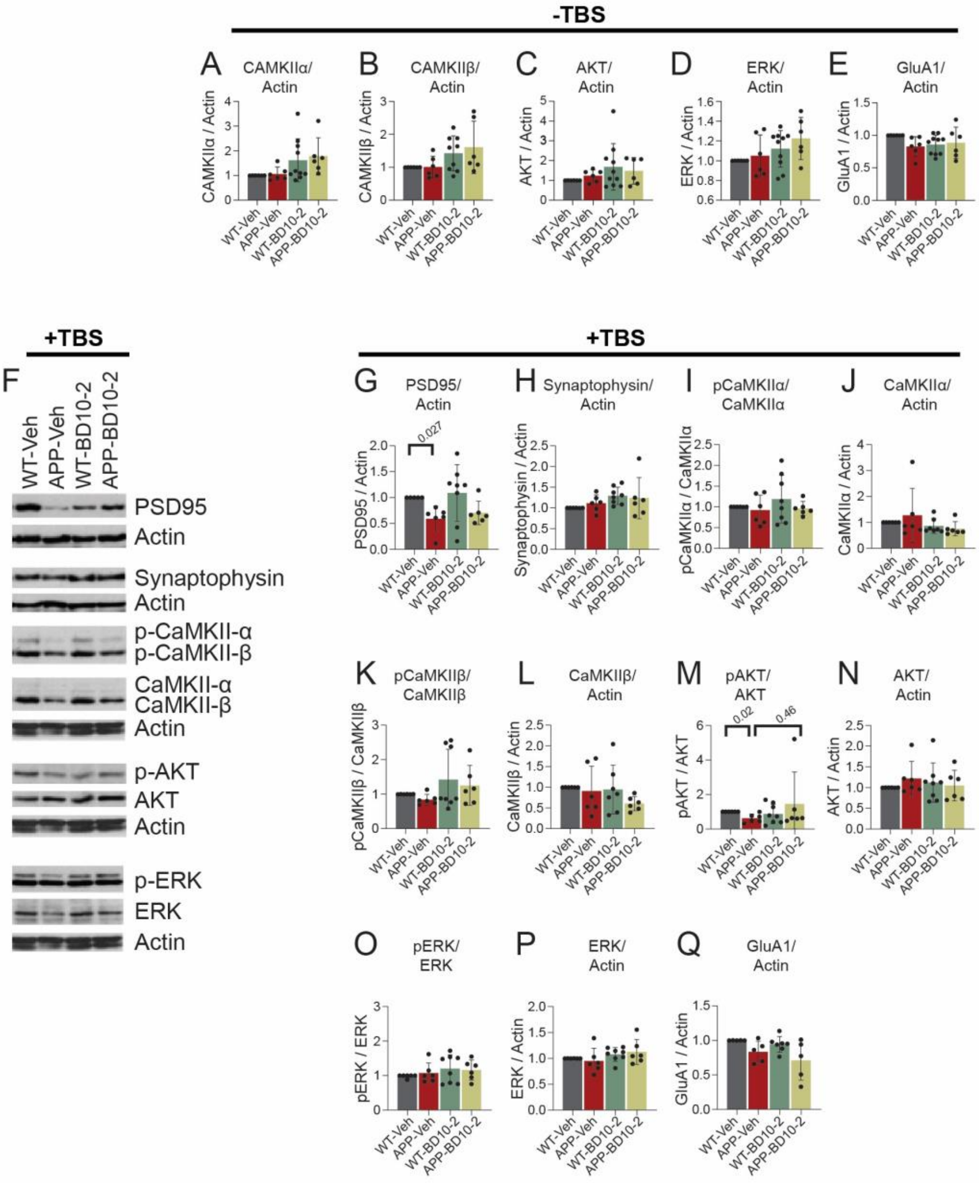

Supplementary Figure 2

Supplementary Fig. 2. **Transcriptomic sample quality control and outlier detection**. **(A)** Heatmap of sample-to-sample Poisson distances clustered hierarchically. **(B)** Standardized sample network connectivity colored by each group. Dashed lines at |Z score| > 2.
