# Supplementary material for: A TrkB and TrkC partial agonist restores deficits in synaptic function and promotes activity‐dependent synaptic and microglial transcriptomic changes in a late‐stage Alzheimer's mouse model
Source: Alzheimers Dement. 2024 May 23;20(7):4434–60. doi: 10.1002/alz.13857 (PMC11247716; doi:10.1002/alz.13857)
Supplement: Supplementary file 3 — Supporting Information [file ALZ-20-4434-s001.pdf]

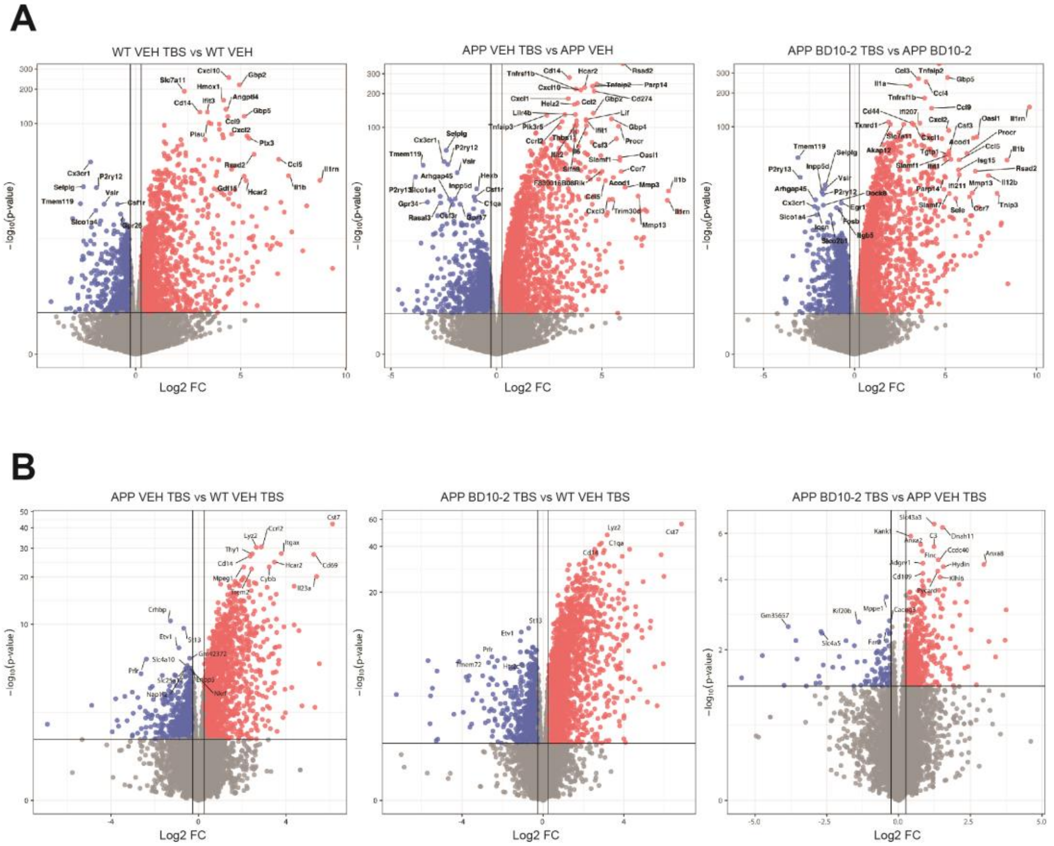

Supplementary Figure 3

Supplementary Fig. 3. **Volcano plots of differential expression (DE) for TBS, APP BD10-2, and APP-BD10-2 effects**. Horizontal lines represent nominal significance of p-value < 0.05, vertical lines indicate fold changes of ± $\log_2(1.2)$. **(A)** Volcano plots examining the TBS effect in experimental groups. *Left:* the TBS effect in WT-Veh mice (WT-Veh-TBS vs WT-Veh); *Center:* the TBS effect in APP-Veh mice (APP-Veh-TBS vs APP-Veh); *Right:* the TBS effect in APP-BD10-2 mice (APP-BD10-2-TBS vs APP-BD10-2). **(B)** Volcano plots of differential expression in stimulated hippocampal slices. *Left:* the APP effect (APP-Veh-TBS vs WT-Veh-TBS); *Center:* the APP-BD10-2 effect (APP-BD10-2-TBS vs WT-Veh-TBS); *Right:* the BD10-2 effect (APP-BD10-2-TBS vs APP-Veh-TBS).
