# Supplementary material for: A TrkB and TrkC partial agonist restores deficits in synaptic function and promotes activity‐dependent synaptic and microglial transcriptomic changes in a late‐stage Alzheimer's mouse model
Source: Alzheimers Dement. 2024 May 23;20(7):4434–60. doi: 10.1002/alz.13857 (PMC11247716; doi:10.1002/alz.13857)
Supplement: Supplementary file 4 — Supporting Information [file ALZ-20-4434-s002.pdf]

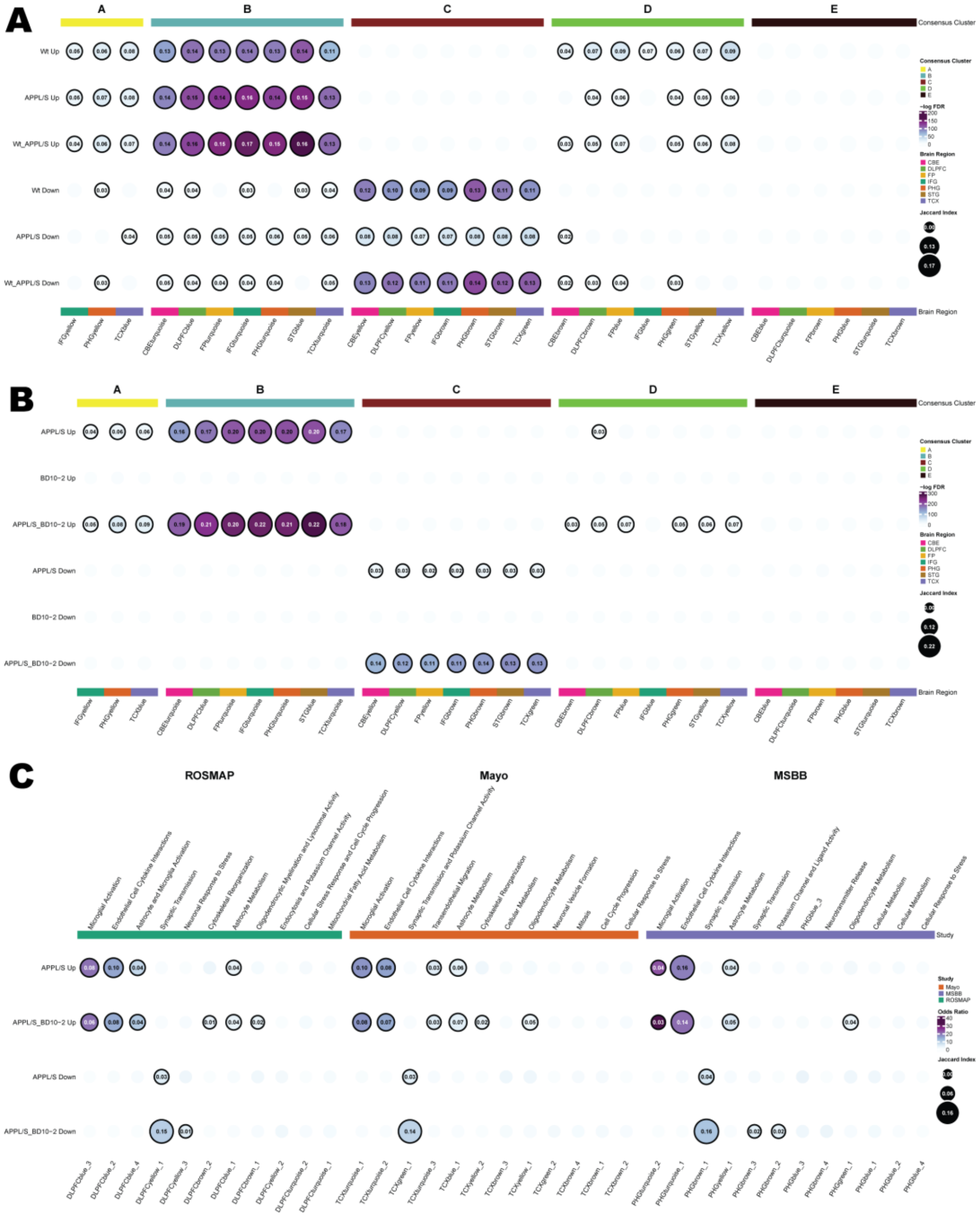

Supplementary Figure 4

Supplementary Fig. 4. **Full overlap enrichment analysis of differential expression datasets**. **(A)** Overlap enrichment (Fisher's exact) of AD-related human-mouse co-expression modules from Wan et al. [52] compared with upregulated (top 3 rows)/downregulated (bottom 3 rows) genes (padj < 0.05) in the TBS effect in the three treatment groups: WT-Veh (WT-Veh-TBS vs WT-Veh), APP-Veh (APP-Veh-TBS vs APP-Veh), and APP-BD10-2 (APP-BD10-2-TBS vs APP-BD10-2). Circles are sized and annotated with the Jaccard index of their overlap and are colored by the significance of the overlap enrichment (-log FDR; circles with an FDR < 0.05 have a bold edge). Modules are grouped based on "consensus clusters", yellow, Astroglial-like modules; light blue, Microglial-like modules; maroon, Neuronal-associated modules; green, Oligodendroglial-like modules. Modules are additionally annotated along the bottom by the associated brain region of the human AD cohort from which they were derived, CBE, cerebellum; DLPFC, dorsolateral prefrontal cortex; FP, frontal pole; IFG, inferior frontal gyrus; PHG, parahippocampal gyrus; STG, superior temporal gyrus; TCX, temporal cortex. **(B)** Overlap enrichment (Fisher's exact) of AD-related human-mouse co-expression modules from Wan et al. [52] compared with upregulated (top 3 rows)/downregulated (bottom 3 rows) DE genes (padj < 0.05) in the APP effect (APP-Veh-TBS vs WT-Veh-TBS), the BD10-2 effect (APP-BD10-2-TBS vs APP-Veh-TBS), and the APP-BD10-2 effect (APP-BD10-2-TBS vs WT-Veh-TBS). **(C)** Overlap enrichment (Fisher's exact) of AD-related human-mouse co-expression modules from Milind et al. [90] compared with upregulated (top 2 rows)/downregulated (bottom 2 rows) DE genes (padj < 0.05) in the APP effect and the APP-BD10-2 effect. Circles are sized and annotated with the Jaccard index of their

overlap and are colored by the odds ratio of the overlap enrichment (circles with an FDR < 0.05 have a bold edge).
