# Supplementary material for: A TrkB and TrkC partial agonist restores deficits in synaptic function and promotes activity‐dependent synaptic and microglial transcriptomic changes in a late‐stage Alzheimer's mouse model
Source: Alzheimers Dement. 2024 May 23;20(7):4434–60. doi: 10.1002/alz.13857 (PMC11247716; doi:10.1002/alz.13857)
Supplement: Supplementary file 5 — Supporting Information [file ALZ-20-4434-s006.pdf]

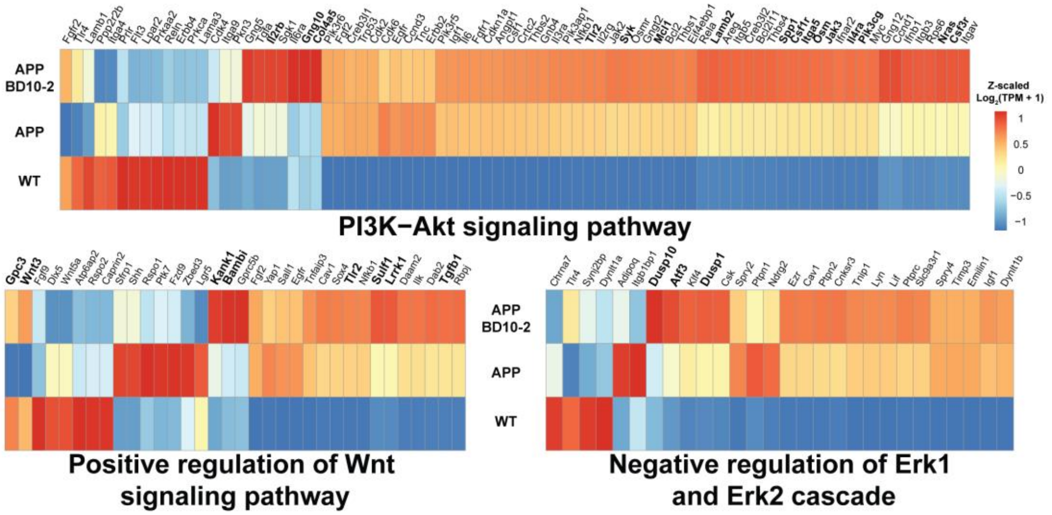

Supplementary Figure 5

Supplementary Fig. 5. **Differential gene expression of neurotrophin modulated enriched pathways in stimulated hippocampal slices from $APP^{L/S}$ mice with or without BD10-2 treatment versus WT-Veh mice.** Examination of gene-scaled expression for genes associated with positive regulation of the Wnt signaling pathway (GO:0030177), PI3K-Akt signaling pathway (KEGG:04151), and negative regulation of ERK1 and ERK2 cascade (GO:0070373). Mean values for each group are Log_2(TPM + 1), then z-scaled across samples. Gene names with nominal significance (p-value < 0.05) in BD10-2 effect (APP-BD10-2-TBS vs APP-Veh-TBS) stimulated slices are bolded.
